# Study of cooling experiment and simulation for edible oil storage

**DOI:** 10.1038/s41598-024-55337-6

**Published:** 2024-02-26

**Authors:** Du Xiao, Chen Yan, Sun Desheng

**Affiliations:** 1 Henan University of Technology, Zhengzhou, 450001 People’s Republic of China; 2 Henan Polytechnic, Zhengzhou, 450018 People’s Republic of China

**Keywords:** Edible oil storage, Thermal stratification, Inner cooling tube, Cooling, CFD, Engineering, Fluid dynamics, Statistical physics, thermodynamics and nonlinear dynamics, Techniques and instrumentation

## Abstract

This paper proposes a refrigerant cooling method using an inner tube in a storage tank to improve the cooling performance and thermal uniformity during the storing of edible oil. With a prototype of an oil tank in Central Grain Reserve of Zhenjiang, the experimental oil tank was built in a scale of 50:1. Both natural and manual cooling experiments were carried out for the experimental tank. The manual cooling process involved two supplying modes for the refrigerant tube (top and bottom) and four different refrigerant temperatures (10 ℃, 12 ℃, 14 ℃, 16 ℃). The experimental results show that, compared with natural cooling, manual cooling can effectively reduce the temperature difference and thermal stratification between upper and lower layers. The temperature difference is 6.79 ℃, 1.93 ℃, and 3.67 ℃ for the natural cooling, manual top supplying, and manual bottom supplying mode, respectively. Furthermore, for the two manual modes, the cooling efficiency of bottom supplying is 21.4% higher than that of the top supplying, and the average oil temperature drops by 0.8–1 ℃. Based on experimental results, different working conditions (20, 40, and 60 ml/s) were simulated to determine the optimal flow rate for bottom supplying mode. The simulation results indicate that the low flow rate (20 ml/s) corresponds to the best thermal uniformity, and the maximum temperature has no obvious change under different flow rate conditions. Therefore, it is not necessary to increase the flow rate to improve cooling efficiency considering the rising energy consumption.

## Introduction

Edible oil is an important component of human diet. It not only provides essential nutrients such as omega-3 and omega-6 fatty acids, but also supplies energy for human activities. As an indispensable part of our daily life, the safe storage of edible oil is particularly important. One of the guiding principles for maintaining the freshness of edible oil is to preserve the stability of its nutritional components. However, the stability of nutritional components is influenced by various factors such as external conditions, internal fatty acids, and enzyme activity^1^. As is well known, the quality of edible oil in storage is a dynamic process. Over time, it gradually deteriorates until it reaches the minimum acceptable standard. Edible oil that falls below the standard becomes inedible, resulting in significant waste of resources^2^. Scholars have been adding synthetic antioxidants to extend the shelf life of edible oils, such as Propyl gallate (PG), butylated hydroxy anisole (BHA), tertiary butylhydroquinone (TBHQ), and butylated hydroxytoluene (BHT). Although these antioxidants can effectively prolong the shelf life, their structural changes are exacerbated under high-temperature reactions, resulting in significant negative impacts on human health and the environment. Therefore, the use of these antioxidants is restricted in many countries^3,4^. Currently, in the absence of significant breakthroughs in additives, scholars have studied the effects of external factors such as temperature, light, and oxygen on the quality of edible oils. The results show that temperature and light conditions have a significant impact on the peroxide value, which is the main factor affecting the quality of edible oils^5–7^. Gargouri et al.^8^ found that oil products packaged in dark glass bottles and metal containers (such as tin cans) have the highest antioxidant stability. As to temperature, it has been found that high temperatures can rapidly increase the peroxide value of oil, accelerate oxidation, and lead to the accumulation of aldehyde substances. On the other hand, low temperatures are more conducive to the stability of the physicochemical properties of oil^9,10^. The recommended temperature for edible oil is 10–25 ℃, and the maximum value should not exceed 40 ℃. As the storage temperature of the oil increases, the peroxide value will also rise to higher than the standard^11^. Mawire A conducted experiments on the cooling process of various edible oils separately. The results showed that the upper layer of oil was cooled at a slower rate than the lower layer, resulting in the phenomenon of thermal stratification^12–14^.

With the deepening of oil storage heat transfer research and the development of computer simulation technology, Zhao et al.^15^ used simulation technology to establish a model of the oil storage tank heating process and found that natural convection-induced plumes play a crucial role in the temperature distribution inside the oil storage tank, while the boundary conditions play a secondary role. Based on this research, Sun et al.^16^ proposed a theoretical model for large tank coil heating process, considering environmental temperature, solar radiation, and oil property parameters. The coupled characteristics of heat transfer and fluid flow were revealed for the heating process of oil, and further investigation was carried out on the influence of different coil structures on temperature distribution and flow patterns^17^. At the same time, the heat transfer, temperature fluctuation characteristics, and solar radiation of large-scale tanks for long-term oil storage have also been included in the scope of research^18^. Solar radiation has an impact on the temperature change of oil, and intensive radiation will cause a rapid oil temperature rise. Therefore, scholars have explored the changes in thermal flux density at different positions of the oil tank through simulation^19,20^. They have established a one-dimensional model^21^ and studied the mechanism of thermal stratification in the storage tank using Gabor finite element method^22^.

Although there have been extensive studies about the influence of temperature on oil storage quality and thermal analysis in oil storage tanks, cooling measures for storage tanks are still relying on natural mode, which leads to poor efficiency and thermal stratification. More effort should be put into the study for efficient cooling system to ensure a favorable storage temperature. Therefore, we proposed an edible oil tank with an embedded refrigerant tube, on which cooling experiments and simulations were conducted to improve the stability of edible oil in the storage tank.

## Experimental system composition and procedure

### Experimental system

**Figure 1:**
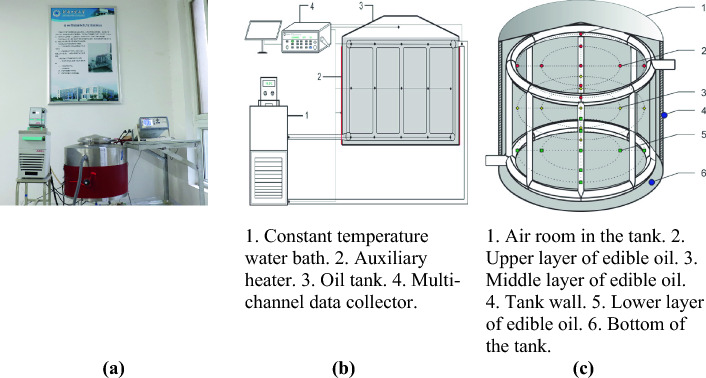
Layout of the experimental device. (**a**) Experimental oil tank. (**b**) Schematic diagram of the experimental system. (**c**) Refrigerant tube and temperature probes layout.

An edible oil storage tank with built-in refrigerant tube was designed to explore the factors affecting thermal stratification and cooling effect of edible oil during storage, as shown in Fig. 1a. The experimental tank was built with reference to a cylindrical steel dome tank in practical oil storage engineering. Its geometric shape and size were designed in a ratio of 50: 1 to the actual one. The cylinder diameter of the experiment tank is 54 cm, the wall height is 42 cm and the arch height is 6.5 cm. An oil inlet was located at the top of the arch, and an oil outlet was located at the bottom of the tank, which can be easily controlled by a valve to facilitate the loading and unloading of oil for experiments. A stripe heater was attached to the outer wall of the storage tank for preheating the oil. Refrigerant solution was provided through a constant temperature water bath (standard heating and cooling thermostatic circulator, JULABO F12-EH). The setup of the experimental system is shown in Fig. 1b. A set of refrigerant tubes were installed inside the storage tank, connected by two DN32 horizontal annular tubes (the upper tube was 33.2 cm above the bottom of the tank, and the lower was 1.5 cm above) and eight DN20 vertical tubes evenly arranged. The layout is shown in Fig. 1c. Temperature probes in the storage tank were arranged at three layers—upper, middle, and lower—positioned at heights of 340 mm, 200 mm, and 60 mm from the bottom of the tank. Nine probes were set on the horizontal plane corresponding to each height. In addition, 3 other probes were placed inside the top of the tank, at the interface between the stripe heater and the tank wall, and at the bottom of the tank to measure the temperature of the air, the stripe heater, and the tank bottom, respectively.

A standard heating and cooling thermostatic circulator (JULABO F12-EH) was used to maintain the temperature of the solution loaded into the refrigerant tubes. During the experiment, two multi-channel temperature collectors were used to monitor the oil temperature and the inlet and outlet temperatures of refrigerant. The specification of instruments used are listed in Table 1.

**Table 1:** Equipment used in the experiment.

Equipment	Quantity	Type	Precision
AnBai Multi-channel temperature acquisition instrument	1	AT4532	± 0.2 ℃
YuWen Single-channel temperature recorder	2	SNN-11E	± 0.1 ℃
Constant temperature water bath	1	JULABO F12-EH	± 0.03 ℃

### Experimental procedure

The experiment was conducted at the Grain and Oil Storage Laboratory of Henan University of Technology in Zhengzhou, China. The experimental oil used was barreled soybean oil. The refrigerant was aqueous solution of ethylene glycol with a water to (CH_2_OH)_2_ volume ratio of 1:1, a density of 1070 kg/m^3^, and a specific heat capacity of 3.2 kJ/(kg ℃). The laboratory temperature was maintained at 22 ± 1 ℃.

The experiment was conducted in three modes: natural cooling, top supplying and bottom supplying modes. In the top supplying mode, the refrigerant entered the upper horizontal tube from the top inlet, being divided by the vertical tubes, then down to the lower horizontal tube, and flowed off the tank from the bottom outlet. In the bottom supplying mode, the flowing direction was opposite. That is, the refrigerant solution entered the oil tank from the bottom inlet, flowed through the lower horizontal tube, branched from vertical tubes, and reached the upper horizontal tube and top outlet.

1. Beforehand, a pre-experiment was introduced to determine the time required for temperature change. It was observed that the most significant change occurred within the first 10 h, while there was virtually no change after 24 h. As a result, the duration of cooling was set to be 24 h. The temperature recorder was programmed to collect data at 5-min intervals.
2. At the beginning, the oil was warmed to a set temperature using the stripe heater. The target average temperature of oil was set at 43 ℃, that is about the maximum temperature occurring in practical engineering. The heating was stopped when the average temperature of the oil reached the target value. This heating procedure was repeated before every cooling began.
3. Next, the experiment was carried out in natural cooling mode. Put the oil in the laboratory environment and let it cool down naturally for 24 h.
4. Then, cooling experiments were conducted using the refrigerant solution with constant temperatures of 10 ℃, 12 ℃, 14 ℃, and 16 ℃ respectively. These cooling experiments were carried out separately for top supplying and bottom supplying mode under each refrigerant temperature. Under these working conditions, the refrigerant flow rate was adjusted to about 45 ± 3 ml/s.

During the experiment, each cooling process was repeated three times to get average values of oil temperature for analysis. The experimental conditions are presented in Table 2 below.

**Table 2:** Experimental scheme.

Flowing direction	Flow rate ml/s	Inlet temperature/℃			
		10	12	14	16
Top suppling	44.37	Scheme 1	Scheme 2	Scheme 3	Scheme 4
Bottom supplying	46.08	Scheme 5	Scheme 6	Scheme 7	Scheme 8

## Edible oil tank cooling experiment

During the process of oil storage cooling, the oil temperature gradually decreased. To compare the cooling efficiency under different operating conditions, the first 600 min of the refrigeration was defined as the high-efficiency stage according to the experimental results. The cooling effects at the end of this high-efficiency stage were thoroughly analyzed.

### Natural cooling experiment of edible oil tank

**Figure 2:**
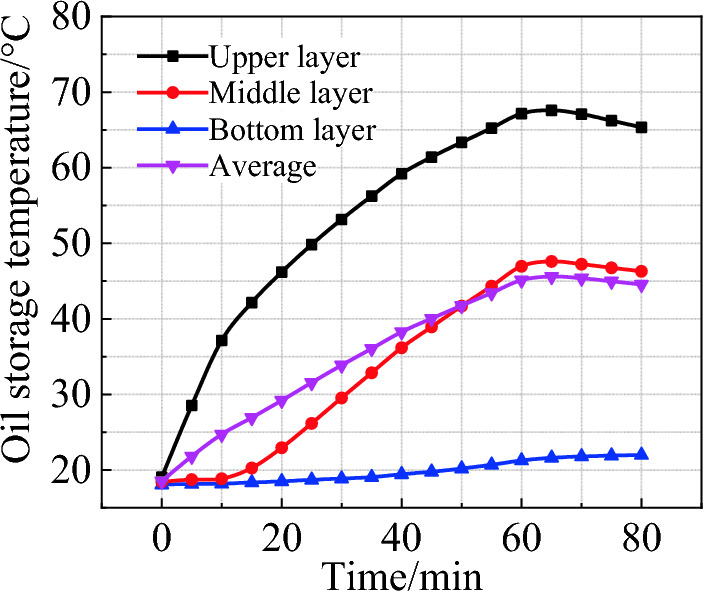
Temperature change during heating.

The 80-min heating process in oil storage is shown in Fig. 2. The final temperature is 65.32 ℃, 46.29 ℃ and 21.99 ℃ for the upper, middle and bottom layer, respectively. The thermal stratification is evident, and the temperature difference between adjacent layers gradually increases with heating time. This is because both the interaction forces between the oil molecules and the density decrease during heating process, which causes the hotter oil to move up and the cooler oil to move down, and results in obvious thermal stratification.

**Figure 3:**
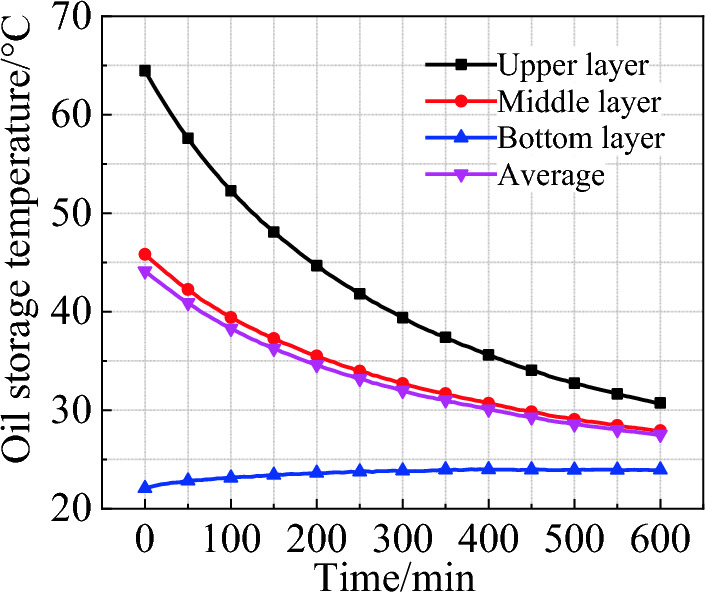
Temperature change during natural cooling.

The 600-min natural cooling process is shown in Fig. 3. The largest temperature drop occurring in the upper layer is 34.59 ℃, and that in the middle layer is 18.37 ℃. However, the temperature of the bottom layer rises slightly because the heat transferred from above is more than that transferred from the bottom to the outside. At the end of the cooling process, the temperature difference between the upper and lower layers is 6.79 ℃, and the overall average oil temperature is 27.53 ℃. The results indicates that the temperature rise mainly occurs in the upper of the oil tank under natural cooling condition, and this should be taken into consideration when designing cooling system.

### Average temperature of the edible oil under top and bottom supplying modes

**Figure 4:**
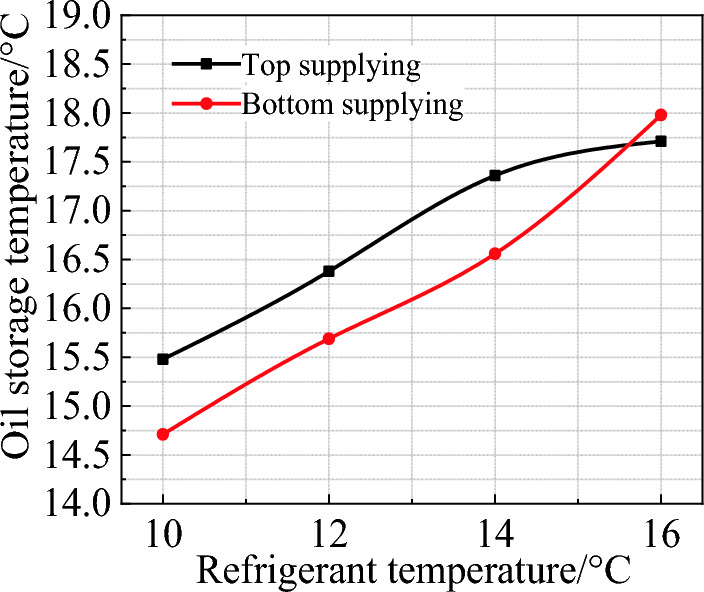
Average oil temperature under different refrigerant flowing modes.

**Figure 5:**
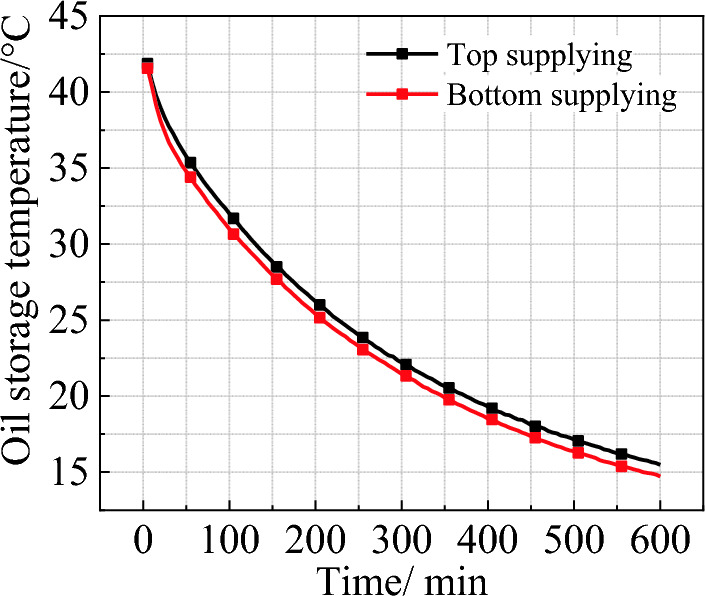
Average oil temperature at refrigerant inlet temperature of 10 ℃.

Figure 4 illustrates the average oil temperature at the end of the high-efficiency cooling stage under different refrigerant temperatures. The results indicate that compared with top supplying mode, the bottom supplying can improve the temperature drop of oil by about 1 ℃ at the refrigerant temperature of 10 ℃, 12 ℃ and 14 ℃. But when the refrigerant temperature is 16 ℃, the average oil temperature in the two modes is almost equal. This means that when the temperature difference between refrigerant and environment is reduced, the difference caused by supplying modes will be weakened. Figure 5 shows the change of the average oil temperature under two supplying modes at a refrigerant temperature of 10 ℃. During the initial cooling stage (within the first 100 min), the cooling efficiency in bottom supplying mode is 0.17 ℃/min, which is 21.4% higher than that of the top supplying mode. After the first 100 min, the average oil temperature in the bottom supplying mode is approximately 0.8–1 ℃ lower compared to the top supplying mode.

### Thermal stratification under different supplying modes

**Figure 6:**
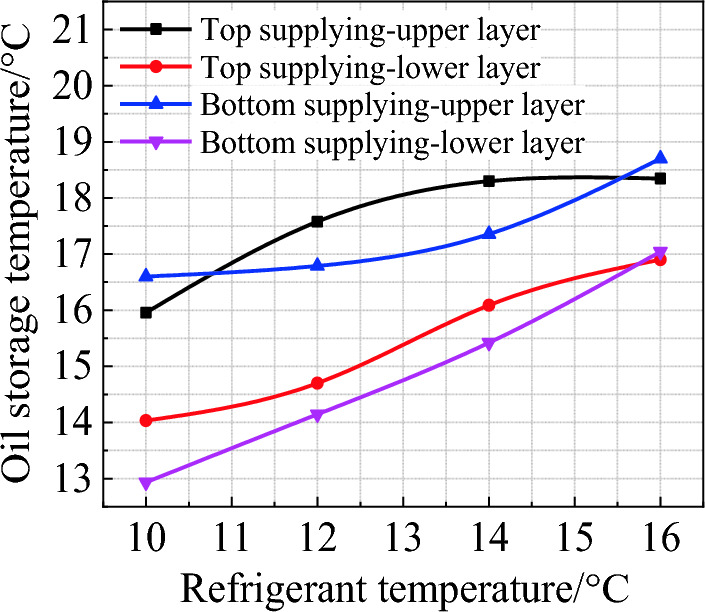
Average oil temperature of upper and bottom layers in the tank at the end of highly effective cooling.

Figure 6 illustrates the change of the average temperature of each layer in the oil tank under two supplying modes. It can be observed that when the refrigerant temperature is 10 ℃, the temperature difference between the upper and lower layers is slightly reduced in the top supplying mode (by about 1.7 ℃). However, when the refrigerant temperature is 12, 14, and 16 ℃, the temperature differences are approximately equal in two modes and even smaller in the bottom mode (by about 0.5 ℃), which indicates a little improved uniformity throughout the tank compared with the top mode. That is, when the refrigerant temperature increases, the top supplying mode produces a larger temperature difference between upper and lower layers compared to the bottom mode, which is detrimental to the stability of oil. Therefore, the bottom supplying mode is preferred for cooling.

**Figure 7:**
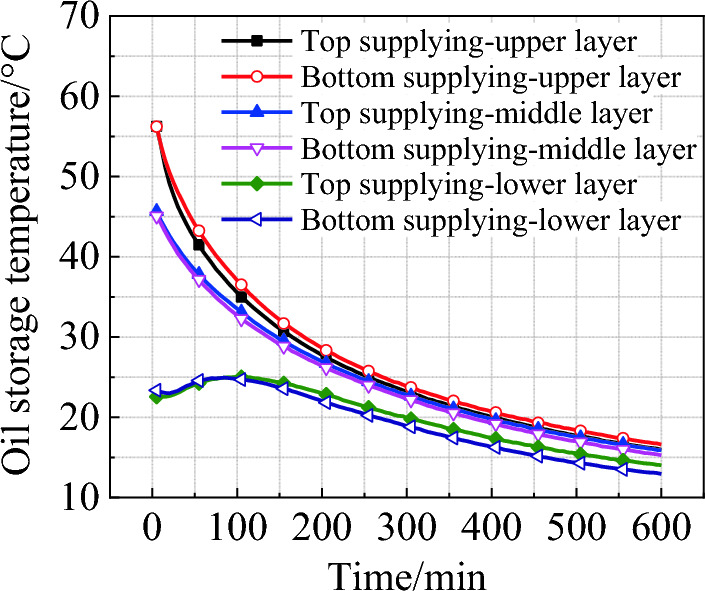
Average oil temperature of each layer in the tank during cooling (refrigerant temperature = 10 ℃).

Figure 7 shows the average temperature of each layer in the oil tank for different supplying modes at a refrigerant temperature of 10 ℃. The temperature curves of the upper and middle layers of the oil show a similar trend in the stage of high-efficiency cooling, and the cooling rate gradually decreases with time. The temperature of the bottom layer rises to 25 ℃ firstly and then gradually decreases. This is because thermal stratification still exists in the initial stage, which leads to the transfer of heat from the upper and middle layers to the bottom, resulting in insufficient cooling provided by the refrigerant. At 100 min, the heat absorbed by the bottom oil reaches a balance with that released to the refrigerant. At the end of this stage, the oil temperatures in the upper and middle layers are approximately equal, but are still 2–2.5 ℃ higher than that of the bottom.

### Analysis of the temperature distribution in different cooling modes

**Figure 8:**
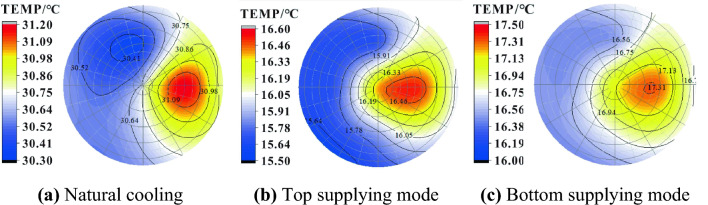
Temperature contour of the upper layer in the tank.

Temperature counter is a useful tool to visualize temperature distribution in different layers and different positions in a cooling system. Figure 8 displays the temperature distribution of oil in the upper layer at a refrigerant temperature of 10 °C for different processes. Due to the refrigerant absorbing heat firstly from the lower layer in the bottom supplying mode, its temperature rises when it goes up into the upper layer and is higher than that of the top supplying mode. The results indicate that the top supplying mode has the highest efficiency, and the average oil temperature is 0.64 °C and 8.01 °C lower than that of the bottom supplying and natural cooling modes, respectively. For all three modes, there is an area on the right side of the cross-section where the temperature is obviously higher than the surrounding area, which is related to the initial thermal stratification and the inlet and outlet locations. The maximum temperature difference decreases in the order of bottom supplying, top supplying and natural cooling modes (1.50, 1.10 and 0.90 °C). Thus, the bottom supplying mode has better temperature uniformity in the horizontal direction than the top supplying.

**Figure 9:**
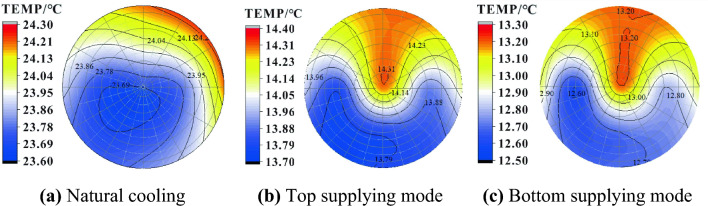
Temperature contour of the lower layer in the tank.

Figure 9 shows the temperature distribution of the lower layer in the oil tank under different supplying modes when the refrigerant is 10 ℃. After 600 min of natural cooling, the average temperature of the lower oil increases by 3.47 °C, which is caused by heat transfer from the upper oil. For the top and bottom supplying modes, the temperature uniformity is similar in the horizontal direction corresponding to a maximum temperature difference of 0.7 and 0.8 ℃, respectively.

According to Figs. 8 and 9, it appears that thermal stratification is significant during natural cooling, with a temperature difference of 6.79 °C between the upper and lower layers. However, refrigerant circulation through the cooling tube eliminates this stratification effectively (top supplying: ∆t = 1.93 °C, bottom supplying: ∆t = 3.67 °C). Furthermore, compared to natural cooling, the manual cooling method provides a larger temperature drop at the lower layer (top supplying: ∆t = 8.52 °C, bottom supplying: ∆t = 10.43 °C). Additionally, a small temperature rise of 1.88 °C occurs in the bottom layer during the natural cooling process because of the heat transferred from above. In terms of cooling efficiency, the bottom supplying mode appears to be superior to the top supplying.

## Numerical simulations on refrigerant cooling for oil tank

Numerical simulations for the oil tank involve using mathematical and computational models to simulate and analyze the behavior of the refrigerant cooling system. This usually includes the use of computational fluid dynamics (CFD) software, such as Ansys, to simulate the flow and heat transfer in the refrigerant tube and oil tank system. The simulation can be used to investigate different operating parameters of the system, such as the refrigerant flow rate and inlet temperature, to optimize the system and improve efficiency.

As mentioned above, at the end of the high-efficiency cooling stage, thermal stratification is observed between the upper and lower layers of the oil. Simulation study was carried out aiming to the effect of the refrigerant flow rate on temperature distribution when the bottom mode is used. To achieve this, a three-dimensional full-size model was established using Gambit 2.4.6. The computational model was then solved using Ansys (Fluent). The simulation field consists of three calculation domains: the air above the oil, the oil, and the refrigerant in the tube. To save the calculation time some reasonable assumptions were adopted to simplify the model:

1. Air is treated as an incompressible fluid.
2. The thermophysical properties of the oil, air, refrigerant, and tank are assumed to be constant.
3. Heat loss at the connection is neglected.

**Figure 10:**
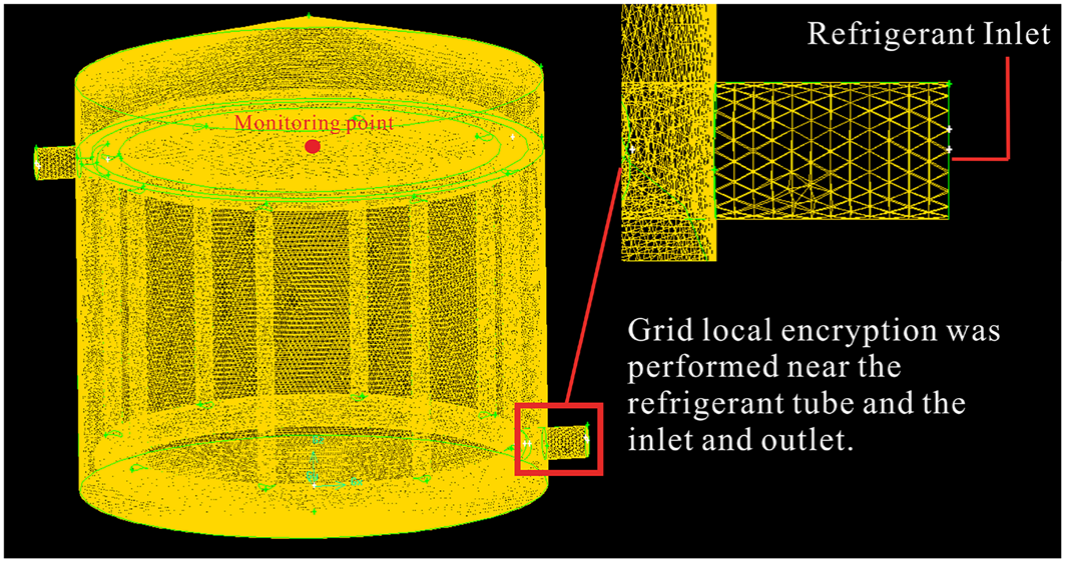
Grid division and meshing.

As shown in Fig. 10, Tet/Hybrid units were utilized in Gambit 2.4.6 to generate an unstructured mesh for the model. Local mesh refinement was employed around the refrigerant and convergence zones. To meet the accuracy requirement, the independence of grid and time step was verified. The center in the upper layer was chosen as the monitoring point. Table 3 shows the influence of three different grid quantities on the calculation. The results indicate that the quantities of 175,000, 245,000, and 315,000 meshes have little effect on the temperature value at the monitoring point with errors in an acceptable range. The time step values were 20s, 60s, and 100s, respectively. The maximum difference at the same time node was 0.2 ℃. When the time step was reduced to less than 60s, no significant improvement was observed in calculation accuracy. Therefore, the simulation was performed using 425,000 meshes and a time step of 60s in Fluent 19.0.

**Table 3:** Comparison of the three kinds of grids.

	Grid1	Grid2	Grid3
Number elements	175,000	245,000	315,000
Final monitoring temperature (℃)	17.707	17.700	17.687
Final error (%)	6.67	6.63	6.55
Calculation time (s)	36,000	36,000	36,000

**Figure 11:**
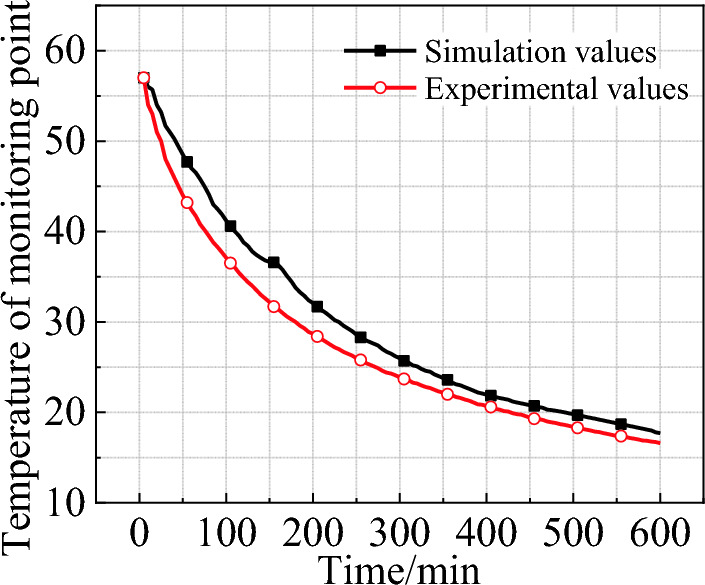
Simulation and experimental values at monitoring point (refrigerant temperature = 10 ℃).

A *k*–*ε* turbulence model was implemented in SIMPLE algorithm solver with a non-stationary, pressure-based and gravity-conscious separated mode. As shown in Fig. 11, the simulation results indicate an average error of 7.6% and a maximum error of 15.4% compared to the experimental data. The main thermophysical properties of the materials involved in the simulations are listed in Table 4.

**Table 4:** Thermal-physical properties in simulation.

Material	Density (kg/m^3^)	Specific heat capacity (J/kg K)	Thermal conductivity (W/m K)	Region properties
Tank (steel)	7850.00	460.00	45.00	Solid
Air	1.23	1006.40	0.024	Fluid
Soybean oil	925	1559.3	0.1275	Fluid
Ethylene glycol solution	1070	3200	0.37	Fluid

**Figure 12:**
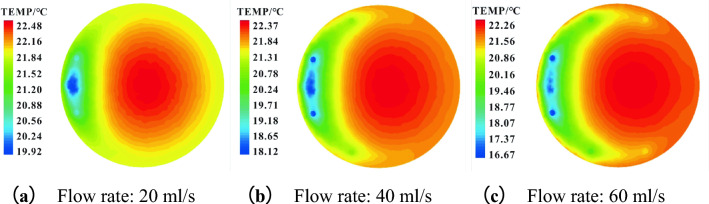
Temperature contour of the lower oil in the tank at different flow rates.

**Figure 13:**
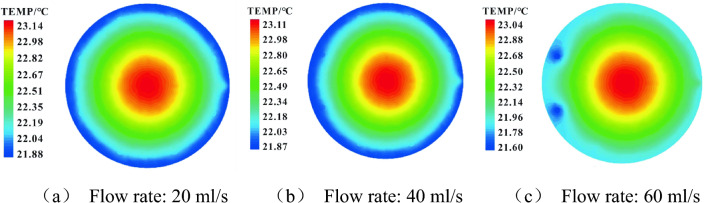
Temperature contour of the upper oil in the tank at different flow rates.

The influence of different refrigerant flow rates (20 ml/s, 40 ml/s, 60 ml/s) on the temperature distribution in the tank was also simulated. The temperature distribution in the lower layer (60mm above the bottom of the tank) and upper layer (340 mm above the bottom of the tank) were presented in Figs. 12 and 13 respectively. As shown in Fig. 12, the minimum value of oil temperature in the lower layer is 19.92 ℃, 18.12 ℃, and 16.67 ℃ for the flow rate of 20 ml/s, 40 ml/s, and 60 ml/s, respectively. However, the difference between the maximum values was negligible, corresponding to oil temperatures of 22.48 ℃, 22.37 ℃, and 22.26 ℃. As flow rate rises, the influenced area in the lower layer expands along the tank wall because of the larger cooling capacity.

As shown in Fig. 13, the minimum temperatures of the upper oil are 21.88 ℃, 21.87 ℃, and 21.60 ℃, respectively, under the flow rate of 20 ml/s, 40 ml/s, and 60 ml/s. The corresponding maximum values are 23.14 ℃, 23.11 ℃, and 23.04 ℃. This means the differences are both small (∆t = 0.28 ℃ and 0.10 ℃, respectively). Increasing the flow rate has no significant influence on cooling efficiency but leads to more obvious thermal stratification. Therefore, more factors should be taken into consideration for the improvement of the whole cooling process.

## Conclusion

The cooling experiment on the edible oil tank shows that, compared to the current common natural cooling, manual cooling with embedded refrigerant tubes can greatly improve the cooling efficiency. With natural cooling, the final temperature of oil is 27.53 ℃, while that of the manual cooling is 15.5 ℃ and 14.7 ℃ for the top and bottom supplying, respectively. Manual cooling can also improve the thermal uniformity of the stored oil. The temperature difference between the upper and lower layers at the end of cooling is 6.79 ℃ under natural mode, obviously larger than that of the top supplying (2.1 ℃) and bottom supplying (3.6 ℃). Although the thermal uniformity of the top supplying mode is slightly better than the bottom supplying, the overall cooling efficiency of the latter is about 21.4% higher than the former in the first 100 min.

Moreover, the numerical simulation results of the bottom supplying mode indicate that with the increase in refrigerant flow rate (20 ml/s, 40 ml/s, 60 ml/s), the cooling effect in the lower layer of the oil tank is significant, but the thermal stratification become more obvious. Large temperature drop occurs in the lower layer and the temperature change in the upper layer remains negligible. Overall, considering the thermal stratification and rising energy consumption associated with the increasing flow rate, a lower value (20 ml/s) is recommended for cooling.

## Data Availability

Due to our laboratory policy, we cannot provide raw data. We have fully described the experimental design, analysis and results, as well as the process of data analysis and processing. But the data used to support the findings of this study are available from the corresponding author upon request.
